# *Lespedeza davurica* (Lax.) Schindl. Extract Protects against Cytokine-Induced *β*-Cell Damage and Streptozotocin-Induced Diabetes

**DOI:** 10.1155/2015/169256

**Published:** 2015-02-22

**Authors:** Bhesh Raj Sharma, Dong Young Rhyu

**Affiliations:** ^1^Department of Oriental Medicine Resources and Institute of Korean Medicine Industry, College of Natural Science, Mokpo National University, 1666 Youngsan-ro, Muan-gun, Jeonnam 534-729, Republic of Korea; ^2^Department of Environmental and Molecular Toxicology, Oregon State University, Corvallis, OR 97331, USA

## Abstract

*Lespedeza *has been used for the management of diabetes in folklore medicine. The purpose of this study is to investigate the protective effects of the methanol extract of* Lespedeza davurica* (LD) on cytokine-induced *β*-cell damage and streptozotocin- (STZ-) induced diabetes. RINm5F cells were treated with interleukin- (IL-) 1*β* and interferon- (IFN-) *γ* to induce pancreatic *β*-cell damage. The exposure of LD extract significantly decreased cell death, nitric oxide (NO) production, nitric oxide synthase (iNOS) expression, and nucleus factor-kappa B (NF-*κ*B) p65 activation. Antidiabetic effects of LD extract were observed by oral glucose tolerance test (OGTT) in normal rats and by checking the biochemical, physiological, and histopathological parameters in STZ-induced diabetic rats. In OGTT, glucose clearance levels improved by oral treatment of LD extract. The water intake, urine volume, blood glucose, and serum TG, TC, TBARS, and DPP-IV levels were significantly decreased, and liver glycogen content was significantly increased by treatment of LD extract (250 mg/kg BW) in STZ-induced diabetic rats. Also, insulin immunoreactivity of the pancreases was increased in LD extract administrated rats compared with diabetic control rats. These results indicate that LD extract may protect pancreatic *β*-cell damage and regulate the blood glucose in STZ-induced diabetic rats.

## 1. Introduction

Type 1 diabetes is characterized by high glucose in the blood. The exact cause of type 1 diabetes is still unknown. But most cases of type 1 diabetes are thought to result from selective destruction of insulin-producing *β*-cells in the islets of Langerhans [1]. During the pathogenesis of type 1 diabetes, infiltration of inflammatory immune cells in and around the islets of Langerhans generates excessive cytokines, including interleukin- (IL-) 1*β*, interferon- (IFN-) *γ*, and tumor necrosis factor- (TNF-) *α* that have been implicated as the effector molecules for the initial destruction of pancreatic *β*-cells [2]. The excessive cytokines upregulate nucleus factor-kappa B (NF-*κ*B) activation, inducible nitric oxide synthase (iNOS) expression, and subsequently NO production, leading to type 1 diabetes [3]. Also, the impaired insulin secretion in type 1 diabetes increases mitochondrial reactive oxygen species (ROS), causing oxidative stress in all tissues [4]. The insulin-secreting *β*-cell itself is the main target of oxidative stress because it contains low levels of antioxidative enzymes [5]. Therefore, protecting number and functions of pancreatic *β*-cells to maintain insulin secretion would help in regulating blood glucose in type 1 diabetes.

Most antidiabetic drugs enhance insulin secretion and decrease insulin resistance to regulate glucose homeostasis throughout the body; however, no medicine is without side effects or entirely safe for long-term use [6]. Thus, many studies have been conducted to find alternative methods for treating diabetic patients [7]. WHO reports that 90% of the world population in rural areas depends on traditional medicine for their primary healthcare and almost 70% of diabetic patients use plants, as a source of medicine, for their primary health needs. However, their use depends largely on ancient practices that have been transmitted from generation to generation, with no or little scientific evidence [8]. Thus, to validate the existing pool of data, there is an urgent need for scientific evidence.

*Lespedeza*, a genus of about 40 species of flowering plants of the pea family, has been used for the treatment and prevention of diabetes in South Korea since ancient times [9]. Several bioactive compounds, including flavonoids, D-fructose, D-pinitol, sterols, and catechins, have been isolated from different species of* Lespedeza *[10]. These compounds have been reported to have antidiabetic activities. Furthermore, we recently discovered the anti-oxidant and anti-diabetic effects of* Lespedeza cuneata* water extract using cell-free systems [11]. Traditional medicine has long been practiced in South Korea for the treatment and prevention of diabetes with plants as an important part of therapy. Korean traditional healers recommend* Lespedeza davurica* (Lax.) Schindl. (LD), locally called “Hopisuri” for treating diabetic patients. Nonetheless, there is a lack of scientific data regarding the effects of* Lespedeza davurica* (Lax.) Schindl. (LD) in diabetes. Thus, to validate existing indigenous knowledge, we asked whether LD has protective effects against cytokine induced *β*-cell damage and action of glucose control in streptozotocin- (STZ-) induced diabetes.

## 2. Materials and Methods

### 2.1. Cell Culture and Reagents

Rat insulinoma RINm5F cells were purchased from the American Type Culture Collection (ATCC) and grown at 37°C under a humidified 5% CO_2_ atmosphere in RPMI 1640 medium (Hycolne), supplemented with 10% FBS, 2 mM glutamine, 100 units/mL of penicillin, 100 *μ*g/mL of streptomycin, and 2.5 *μ*g/mL of amphotericin B. IL-1*β* and IFN-*γ* were purchased from R&D Systems (Minneapolis, MN). STZ, tolbutamide (TLB), and carboxymethylcellulose (CMC) were purchased from Sigma-Aldrich (St. Louis, MO, USA). All chemicals and reagents were of analytical grade.

### 2.2. Preparation and Analysis of LD Extract

LD was collected in Jeonnam province, South Korea. The taxonomic identity of the plant was confirmed by Professor Hui Kim, and the sample was preserved for reference in the herbarium of the Department of Oriental Medicine Resources, Mokpo National University, South Korea. The whole plant was dried in an oven at 40°C and then was powdered and extracted with 80% methanol, followed by filtration, vacuum evaporation, and freeze drying. The yield of the extract was about 8% of the starting material. The concentration of total flavonoids and polyphenols content in LD extract and the presence of quercetin and different catechins in the extract were measured by colorimetric analysis as described previously [12].

### 2.3. *In Vitro* Assays

#### 2.3.1. Cell Viability

Briefly, RINm5F cells were seeded in 96-well plates (2 × 10^5^ cells/well) and left overnight to adhere. After the attachment of cells in the plates, LD extract was added for 3 h, and then cytokines were added for 48 h. The cells were then washed twice with PBS and MTT solution (1 mg/mL) was added to each well, followed by incubation at 37°C for 1 h. Finally, DMSO was added to dissolve the formazan crystals. The optical density was then measured at 540 nm using a spectrophotometer (Immuno Mini NJ-2300, Japan).

#### 2.3.2. Measurement of NO Production

Briefly, following treatment with sample and cytokines for 48 h, 100 *μ*L aliquots of the culture supernatants were incubated with 100 *μ*L of a 1 : 1 mixture of 1% sulfanilamide in 5% phosphoric acid and 0.1% N-(1-naphthyl) ethyldiamine dihydrochloride, at room temperature for 5 min. Then, the optical density at 540 nm was measured using the spectrophotometer (Immuno Mini NJ-2300). The concentrations of NO were then calculated by a linear standard curve generated from serial dilutions of sodium nitrite in working medium.

#### 2.3.3. Western Blot Analysis

RINm5F cells treated with cytokines were placed in an ice-cold lysis buffer (PRO-PREP; iNtRON Biotechnology, Korea) for 10 min to extract proteins. Then, lysed cells were centrifuged (13,000 rpm, 20 minutes, 4°C), and the supernatant was collected as the lysate. To analyze for the translocation of NF-*κ*B p65 subunit, the cells were stimulated with cytokines for 25 min and cytosolic and nuclear fractions were prepared using the NE-PER kit (Thermo Scientific, USA). Lysate containing 30 *μ*g of protein was separated by 10% SDS-PAGE. It was then transferred to the nitrocellulose sheet in a western blot apparatus (Bio-Rad, Hercules, CA). The nitrocellulose sheet was blocked with 5% skim milk for 1 h, followed by TBS-T (1 M Tris, 5 M NaCl, and 0.1% Tween 20) washing, and was incubated for 2 h with primary antibodies, iNOS (1 : 500, Santa Cruz Biotechnology, USA) or NF-*κ*B p65 (1 : 500, Santa Cruz Biotechnology, USA). Then, the nitrocellulose membrane was again washed with TBST followed by incubation with the secondary antibody (horseradish peroxidase-conjugated IgG, 1 : 2,000) for 1 h. Then, the expressed proteins were measured by analyzing the signal captured on the nitrocellulose membrane using an image acquisition and analysis software (Visionworks LS; UVP, LLC).

#### 2.3.4. RNA Isolation and Reverse Transcription Polymerase Chain Reaction (RT-PCR) for iNOS Expression

Total RNA was isolated from cells using the TRI reagent (Molecular Research Center, Inc.). Purification and quantification of isolated RNA were assessed by measuring the optical density at 260 and 280 nm in a nanodrop spectrophotometer (Thermo Scientific, Waltham, MA, USA). For rat iNOS, 300 ng of isolated RNA was added to a final 30 *μ*L volume of Diastar2x One-Step RT-PCR premix kit with each primer, forward primer, 5′-GGTCCAACCTGCAGGTCTTC-3′, reverse primer, 5′-GGTCCATGATGGTCACATTC-3′. The temperature cycle for the PCR reaction was 50°C for 30 min, 95°C for 15 min, and 35 cycles of amplification (denaturation at 95°C for 20 s, annealing at 59°C for 40 s, and extension at 72°C for 1 min). After the last cycle, the samples were incubated for a further 5 min at 72°C. *β*-actin PCR was performed as a control with upstream primer 5′-TGCCCATCTATGAGGGTTACG-3′ and downstream primer 5′-TAGAAGCATTTGCGGTGCACG-3′. The obtained PCR products were analyzed on RedSafe (iNtRON Biotechnology, Korea) stained 1.5% agarose gel using an image acquisition and analysis software (Visionworks LS; UVP, LLC).

### 2.4. *In Vivo* Assays

#### 2.4.1. Experimental Animals

Healthy male Wistar rats (180–220 g), purchased from the Central Lab Animal Inc. (Seoul, Korea), were maintained under standard light (12/12-h light/dark) and temperature conditions (22 ± 2°C). All rats were provided with standard pellet diet* ad libitum* and had free access to water. The animals were acclimatized to the laboratory conditions during 1 week prior to the commencement of the experiment. Procedures involving the animal care were conducted in conformity with the institutional guidelines of Mokpo National University, Korea.

#### 2.4.2. Oral Glucose Tolerance Test (OGTT)

LD extract and TLB (250 mg/kg BW) were administered 30 min prior to glucose loading (2 g/kg BW) in overnight-fasted rats, which were divided into three different groups. Control group received only saline solution. Then, blood glucose levels were measured at 0, 30, 60, 90, and 120 min using a blood glucose test meter (GlucoDr, Allmedicus, Korea), respectively.

#### 2.4.3. Experimental Induction of Diabetes

Diabetes was induced in overnight-fasted rats by a single intravenous injection of STZ (50 mg/kg BW) freshly dissolved in cold 0.1 M citrate buffer (pH 4.5). Control rats received only citrate buffer. The fasting blood glucose level of rats was tested for successful induction of diabetes at 72 h after STZ induction. Only those rats having blood glucose level >250 mg/dL were used in the study.

#### 2.4.4. Experimental Design

Rats were assigned randomly into four groups of eight animals each. Group of control and STZ received only vehicle (0.1% CMC). Each sample was dissolved in the vehicle and was administered at 250 mg/kg BW once daily for 4 weeks.

 Group I (control) served as normal rats with treatment of 0.1% CMC.
 Group II (STZ) served as STZ-induced diabetic rats with treatment of 0.1% CMC.
 Group III (STZ + LD) served as STZ-induced diabetic rats with treatment of LD extract (250 mg/kg BW).
 Group IV (STZ + TLB) served as STZ-induced diabetic rats with treatment of TLB (250 mg/kg BW).

Body weight, dietary intake, and water intake were measured twice per week during the experimental period. At the end of the experiment, overnight-fasted rats were anesthetized with ether and blood was collected from the abdominal artery for various biochemical analyses. Kidneys and livers were removed surgically, and their weights were measured. Urine volume was calculated, one day before fasting for 24 h.

#### 2.4.5. Measurement of Blood Glucose Level

Blood was collected from the tail vein of overnight (10–12 h) fasted rats in each week for the determination of blood glucose, using a blood glucose test meter (GlucoDr, Allmedicus, Korea).

#### 2.4.6. Measurement of Serum Triglycerides (TG) and Total Cholesterol (TC) Levels

Blood was centrifuged (3000 rpm, 20 min, 4°C) for separating serum. The levels of serum TG and TC were measured spectrophotometrically using commercially available kits (Asan Pharmaceutical Company, Seoul, Korea).

#### 2.4.7. Measurement of Serum Dipeptidyl Peptidase- (DPP-) IV and TBARS Levels

To measure serum DPP-IV activity, an aliquot of serum (10 *μ*L) was incubated for 60 min at 37°C with 50 *μ*L of 50 mM HEPES buffer (pH 7.4) containing the Gly-Pro-AMC substrate (final substrate concentration, 1 mM). The reaction was stopped by the addition of 3 M acetic acid. Then, released AMC was measured using a Perkin Elmer Victor^3^V 1420 Multilabel Plate Counter (Perkin Elmer, USA) at an excitation wavelength of 370 nm and an emission wavelength of 440 nm. Serum TBARS level was measured according to the method described previously [13].

#### 2.4.8. Measurement of Liver Glycogen and TBARS Levels

Hepatic tissues were homogenized in hot ethanol (80%) at a concentration of 100 mg/mL and then centrifuged (9500 rpm, 20 min). The residue was collected, dried over a water bath, and then extracted at 0°C for 20 min by adding a mixture of 5 mL water and 6 mL of 52% perchloric acid. The collected material was centrifuged at 9500 rpm for 15 min to recover the supernatant. Next, 0.2 mL supernatant was transferred into a graduated test tube and made to 1 mL volume by the addition of distilled water. Then, 4 mL of anthrone reagent was added to all the test tubes. The test tubes were then heated in a boiling water bath for 8 min, allowed to cool to room temperature, and the intensity of the green to dark green color of the solution was measured at 630 nm. Glycogen content of the sample was determined from a standard curve prepared with a standard glucose solution. Liver glycogen was measured according to the method described by Gutierrez et al. [14].

#### 2.4.9. Histology and Immunohistochemistry (IHC)

The pancreases were fixed in the 10% neutral buffered formalin processed routinely and embedded in paraffin wax. The sections of pancreas were cut at 4 *μ*m thick. The sections were stained with hematoxylin and eosin (H&E). For IHC, the sections were incubated in a solution of 3% hydrogen peroxide prepared in methanol for 40 min and microwaved at 750 W for 10 min in 0.01 mol/L citrate buffer. After being blocked with rabbit serum (Vector Laboratories, Burlingame, CA, USA) for 30 min, the tissue sections were immunostained with the primary antibody, a rabbit monoclonal anti-insulin (1 : 200, Santa Cruz Biotechnology, USA). The antigen-antibody complex was visualized by an avidin-biotin peroxidase complex solution using an ABC kit (Vector Laboratories, Burlingame, CA, USA). The sections were rinsed in distilled water and counterstained with Mayer's hematoxylin.

### 2.5. Statistical Analysis

The SPSS software (SPSS, Inc., Chicago, USA) was used for statistical analyses. All data are presented as means ± SE. Groups were compared using ANOVA, followed by Duncan's* post hoc *test of multiple comparisons. *P* values < 0.05 were considered to indicate statistical significance.

## 3. Results

### 3.1. Components of LD Extract

LD powder was prepared as described under Section 2. LD extract showed high polyphenol (65 mg equivalent of gallic acid/g of the extract) and flavonoids (80 mg equivalent of rutin/g of the extract) content. The spectrophotometric analysis of the extract showed the presence of quercetin, catechin, epicatechin, and epigallocatechin gallate.

### 3.2. Effect of LD Extract on IL-1*β*- and IFN-*γ*-Induced Cell Death in RINm5F Cells

**Figure 1:**
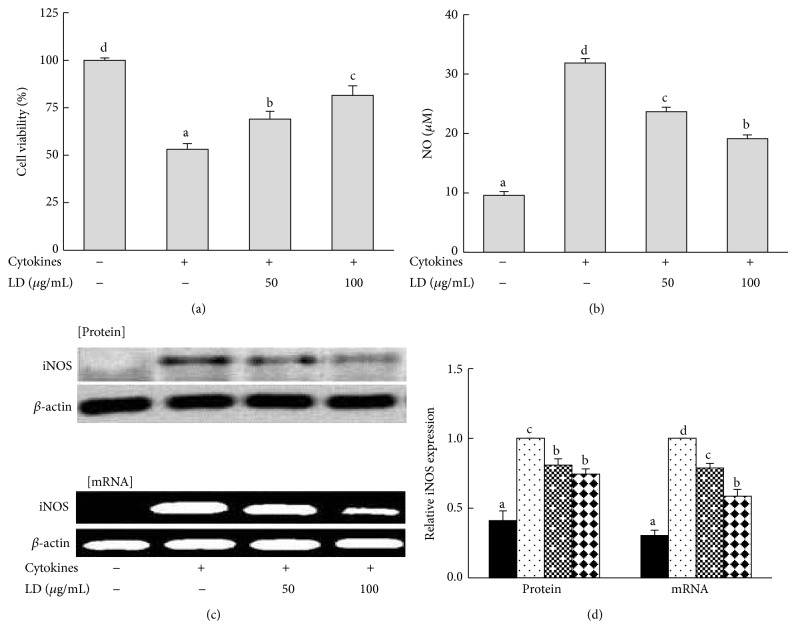
Effects of LD extract on cytokine-induced cell death, NO production, and iNOS protein and mRNA expression in RINm5F cells. RINm5F cells (2 × 10^5^) were pretreated with the indicated concentrations of LD extract for 3 h, followed by stimulation with IL-1*β* (2 ng/mL) and IFN-*γ* (100 U/mL) for 48 h. Cell viability (a), NO production (b), and iNOS protein and mRNA expression (c) were determined by the MTT assay, Griess reagent, western blotting, and RT-PCR analyses, respectively. Each value represents the mean ± SE of three independent experiments. Bars with different letters are significantly different at *P* < 0.05 (Duncan's multiple comparison tests).

RINm5F cells were treated with various concentrations of LD extract to assess its cytotoxicity. Then, nontoxic doses of LD (50 and 100 *μ*g/mL) were used for further experiments. As shown in Figure 1(a), the combination of IL-1*β* and IFN-*γ* decreased cell viability to 53%. However, the addition of LD extract (50 and 100 *μ*g/mL) increased cell viability to 69 and 81%, respectively.

### 3.3. Effects of LD Extract on IL-1*β*- and IFN-*γ*-Induced NO Production and iNOS Expression in RINm5F Cells

The mixture of IL-1*β* and IFN-*γ* induced pancreatic *β*-cell death via NO production and iNOS expression. As shown in Figure 1(b), incubation of RINm5F cells with IL-1*β* and IFN-*γ* for 48 h showed a significant increase of NO production to 69.1% compared with nontreatment of cytokines. However, the addition of LD extract (50 and 100 *μ*g/mL) significantly decreased NO production to 25.4 and 39.9% in cytokine-induced RINm5F cells, respectively. Also, the expression of iNOS mRNA and protein was inhibited dose-dependently by treatment with 50 and 100 *μ*g/mL LD extract (Figure 1(c)).

### 3.4. Effect of LD Extract on the Activation of IL-1*β*- and IFN-*γ*-Induced NF-*κ*B p65 Subunit in RINm5F Cells

**Figure 2:**
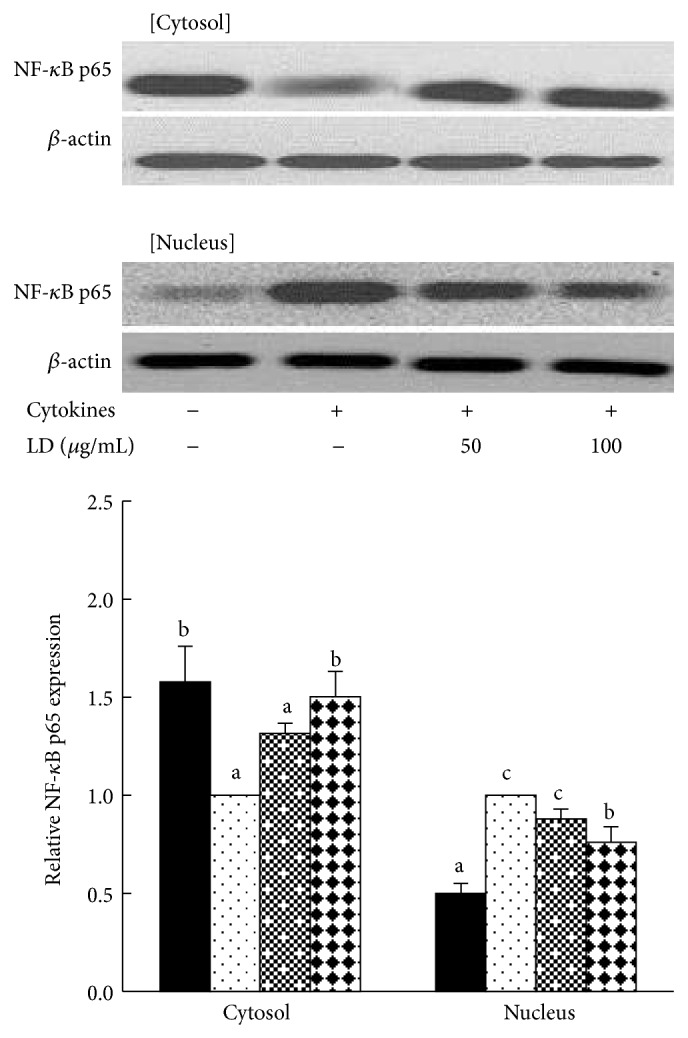
Effects of LD extract on the activation of cytokine-induced NF-*κ*B p65 subunit in RINm5F cells. RINm5F cells (2 × 10^5^) were pretreated with the indicated concentrations of LD extract for 3 h, followed by stimulation with IL-1*β* (2 ng/mL) and IFN-*γ* (100 U/mL) for 25 min. The activation of NF-*κ*B p65 subunit was determined by western blotting, by measuring the expression of NF-*κ*B p65 subunit in both the cytosolic and nuclear compartments. Each value represents the mean ± SE of three independent experiments. Bars with different letters are significantly different at *P* < 0.05 (Duncan's multiple comparison tests).

NF-*κ*B plays an important role in transcriptional regulation of cytokine-induced *β*-cell damage. As shown in Figure 2, IL-1*β*- and IFN-*γ*-induced RINm5F cells significantly increased the activation of NF-*κ*B p65 subunit into the nuclear compartment, enhancing its translocation from the cytosol to the nucleus. However, the treatment of LD extract highly increased expression of NF-*κ*B p65 subunit in cytosolic compartment, preventing its translocation from the cytosol to the nucleus.

### 3.5. Effect of LD Extract on OGTT in Normal Rats

**Figure 3:**
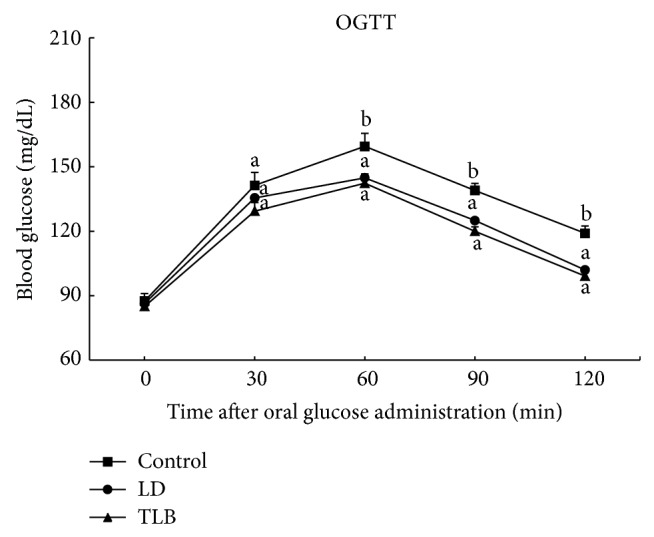
Effect of LD extract on OGTT in normal rats. Values are given as means ± SE for each group of eight animals. Bars with different letters are significantly different (*P* < 0.05, Duncan's multiple comparison tests). LD (*L. davurica*) and TLB (tolbutamide) were administered at a dose of 250 mg/kg BW.

OGTT was performed to evaluate how quickly blood glucose is cleared from blood by LD extract. After oral administration of glucose in normal rats, blood glucose levels were significantly higher at 60 min. The administration of LD extract and TLB at the dose of 250 mg/kg BW exhibited 14.2 and 16.8% reduction in blood glucose compared with saline treated group at 120 min (Figure 3).

### 3.6. Effect of LD Extract on Blood Glucose Levels in STZ-Induced Diabetic Rats

**Figure 4:**
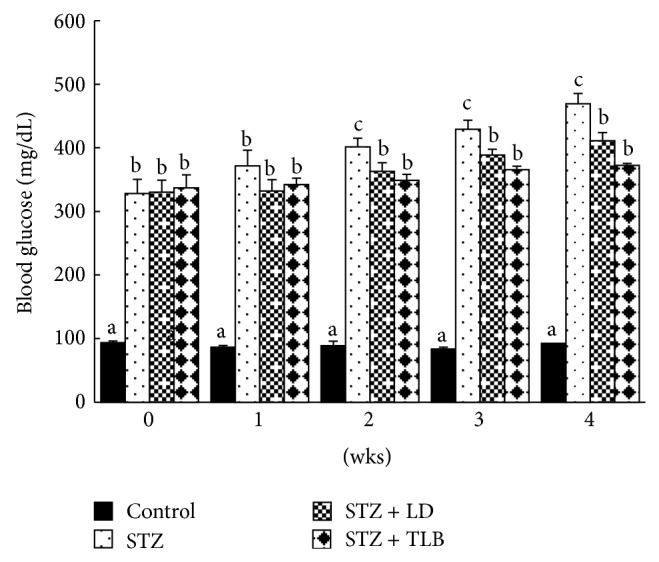
Effect of LD extract on blood glucose in STZ-induced diabetic rats. Values are given as means ± SE for each group of eight animals. Bars with different letters are significantly different (*P* < 0.05, Duncan's multiple comparison tests). LD (*L. davurica*) and TLB (tolbutamide) were administered at a dose of 250 mg/kg BW for 4 weeks.

As shown in Figure 4, the blood glucose level increased continuously in the STZ-induced diabetic group during the experimental period. However, the administration of LD extract tended to decrease significantly (*P* < 0.05) blood glucose levels from second week of the experiment. In the fourth week, LD treated group significantly decreased blood glucose to 12.3% (from 469 ± 16 to 411 ± 13 mg/dL) compared to diabetic group, whereas TLB as standard drug effectively decreased blood glucose by 24% (from 469 ± 16 to 373 ± 3 mg/dL).

### 3.7. Effects of LD Extract on Body Weight, Liver Weight, Kidney Weight, Dietary Intake, Water Intake, and Urine Volume

Body weight increased continuously in the control group and decreased in STZ-induced diabetic group during the experimental period (Table 1). However, the LD extract-treated group showed a significant gain in body weight at the end of experiment compared with the STZ-induced diabetic group. No significant change was found in the liver or kidney weights of the STZ-induced diabetic group or LD extract- or TLB-treated groups. The ascending dietary intake, water intake, and urine volume in STZ-induced diabetic rats were significantly decreased by oral administration of LD extract or TLB.

### 3.8. Effects of LD Extract on Serum TC, TG, TBARS, and Liver TBARS and Glycogen Levels

As shown in Table 2, the levels of serum TC, TG, and TBARS and liver TBARS showed a significant increase, and liver glycogen significantly decreased in STZ-induced diabetic rats. However, the oral administration of LD extract significantly decreased the levels of serum TC (35.7%), TG (33.6%), TBARS (25.6%), and liver TBARS (30.8%) and increased liver glycogen level by 31.1% compared with STZ-induced diabetic rats, respectively. TLB, an antidiabetic drug, was more effective than LD extract.

### 3.9. Effect of LD Extract on Serum DPP-IV Level in STZ-Induced Diabetic Rats

**Figure 5:**
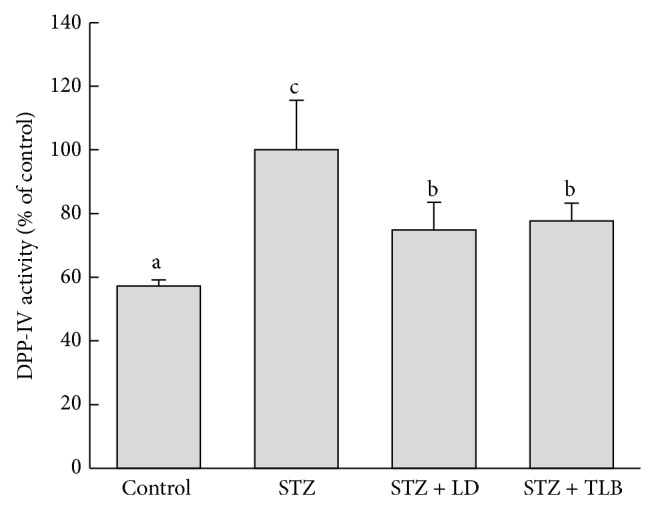
Effects of LD extract on DPP-IV in STZ-induced diabetic rats. Values are given as means ± SE for each group of eight animals. Bars with different letters are significantly different (*P* < 0.05, Duncan's multiple comparison tests). LD (*L. davurica*) and TLB (tolbutamide) were administered at a dose of 250 mg/kg BW for 4 weeks.

As shown in Figure 5, STZ caused a significant increase in activity of serum DPP-IV enzyme by 57.3%, when compared with control rats. Oral administration of LD extract significantly decreased DPP-IV activity by 25.1% compared with diabetic rats. The effect of LD extract on DPP-IV activity was comparable to those of the reference drug, TLB.

### 3.10. Effects of LD Extract on Histology and Immunohistochemistry of Pancreatic Islets in STZ-Induced Diabetic Rats

**Figure 6:**
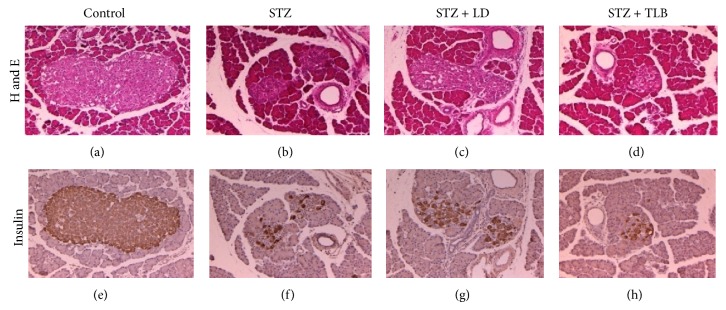
Immunohistochemical (IHC) and H&E staining of pancreatic islets in STZ-induced diabetic rats. Pancreases were obtained from control ((a), (e)), diabetic control ((b), (f)), LD-treated diabetic group ((c), (g)), and TLB-treated diabetic group ((d), (h)). Islets and adjoining exocrine regions were stained with H&E ((a)–(d)). Islets were labeled with an anti-insulin antibody and peroxidase-labeled anti-rabbit IgG ((e)–(h)). Scale bar is 100* μ* m. LD (*L. davurica*) and TLB (tolbutamide) were administered at a dose of 250 mg/kg BW for 4 weeks.

The effects of LD extract on pancreatic *β*-cell damage in STZ-induced diabetic rats were examined histologically. Pancreatic tissues were subjected to H&E staining and immunohistochemistry. STZ-treated rats showed degenerative and necrotic changes and islet shrinkage as well as weak insulin reactivity. However, LD extract-treated tissues showed round and clearly defined islets that were strongly positive for insulin (Figure 6).

## 4. Discussion

Medicinal plants have been widely used for the treatment and prevention of diabetes. However, their scientific evidences are not yet fully understood and need to be better explored. In the present study, we investigated the effects of LD extract on pancreatic *β*-cell damage and glucose regulation using cellular and animal models of type 1 diabetes. We found that LD extract protected *β*-cell damage in cytokine-mediated RINm5F cells, and its mechanism was associated with the inhibition of NF-*κ*B p65 activation. In STZ-induced diabetic rats, the oral administration of LD extract significantly decreased blood glucose and DPP-IV activity and increased the liver glycogen content. An increased mass of *β*-cells was observed in pancreatic islets of LD-administered rats. Thus, this study proved the scientific evidence for traditional use of LD in diabetes.

Type 1 diabetes is caused when an excessive immune response selectively destroys insulin-producing *β*-cells. During the pathogenesis of type 1 diabetes, pancreatic *β*-cells generate inflammatory cytokines that increase NO production and cause pancreatic *β*-cell death [15]. IL-1*β* and IFN-*γ* as inflammatory cytokines highly decreased cell viability of pancreatic *β*-cells by activating NF-*κ*B translocation [3]. The role of NF-*κ*B, which can be activated by cytokines in type 1 diabetes, has been implicated as key signaling mediator of NO production or dysfunction and destruction of pancreatic *β*-cells [16]. We measured the effects of LD extract on IL-1*β*- and IFN-*γ*-induced *β*-cell damage in RINm5F cells. LD extract showed protective effects against cytokine-induced *β*-cell damage and significantly decreased NO production and iNOS expression. The inhibitory effect of LD extract on cytokine-induced *β*-cell damage was related to the suppression of NF-*κ*B p65 translocation from cytosol to nucleus. Therefore, LD extract could protect pancreatic *β*-cell damage through regulation of NF-*κ*B p65 in response to cytokine-induced *β*-cell damage.

STZ-mediated type 1 diabetes is caused primarily by the selective destruction of insulin-secreting *β*-cells with excessive release of NO production, and its consequence elevated blood glucose in the body [17]. In our results, LD extract not only significantly improved the glucose utilization in STZ-induced diabetic rats, but also increased glucose clearance in normal rats. However, LD extract showed lesser effects on glucose metabolism and urinary excretion compared with TLB treated rats. This might be associated with the relationship between insulin secretion and urinary excretion. Although phenolic compounds contained in LD have antidiabetic effects, some study suggested that pinitol in LD has no effect on insulin-mediated glucose disposal due to abnormality of insulin-signaling pathway in diabetes [18]. Pinitol, which has an insulin-like activity, is found in the species of* Lespedeza*. We speculated that some components in LD may protect the function and mass of pancreatic *β*-cells and enhance insulin levels without a proportional impact on fasting glucose.

In type 1 diabetes, the hepatic tissue cannot synthesize glycogen due to defective glucose mobilization as a result of the decreased insulin secretion. The unavailability of carbohydrates as an immediate energy source causes the breakdown of tissue proteins. Thus, body weight loss is seen in type 1 diabetes. DPP-IV inhibitors have been recently developed as a new class of drugs for the treatment of diabetes, because DPP-IV inhibits glucose metabolism by degrading incretin hormones that play an important role in insulin secretion [19]. In this study, LD extract not only increased body weight and glycogen content of the liver, but also inhibited DPP-IV activity in STZ-induced diabetic rats. Our results indicated that LD extract could control the glucose metabolism via protective effect of insulin-secreting *β*-cells.

LD is a rich source of flavonoids and polyphenols; it contains kaempferol, tamarixetin, leteolin, quercetin, epicatechin, triofolin, rutin, and some phenolic compounds [20]. The antidiabetic activity of flavonoids and polyphenols has recently been reported [21]. We found high polyphenol (65 mg equivalent of gallic acid/g of the extract) and flavonoid (80 mg equivalent of rutin/g of the extract) content in the LD extract. Thus, we speculate that flavonoids and polyphenols in LD extract may also be involved in its antidiabetic activities. Based on this study, LD should be considered as a useful candidate of folk medicine for the treatment and prevention of diabetes.

## 5. Conclusion

This study is the first to demonstrate that LD has a *β*-cell protective effect and can regulate blood glucose in type 1 diabetes. The antidiabetic effects of LD may be due to the protection of pancreatic *β*-cell function via down regulation of transcription factor NF-*κ*B in cytokine-induced *β*-cell damage, and the regulation of blood glucose in STZ-induced diabetic rats. Thus, our results scientifically support the folkloric use of LD as hypoglycemic agent.

## Figures and Tables

**Table 1:** Effect of LD extract on body weight, liver weight, kidney weight, diet intake, water intake, and urine volume in STZ-induced diabetic rats.

Group	Body weight (g)		Liver weight	Kidney weight	Diet intake	Water intake	Urine volume
	Initial	Final	(g/100 g BW)	(g/100 g BW)	(g/day)	(mL/day)	(mL/day)
Normal	238 ± 2	350 ± 7^c^	2.63 ± 0.07^a^	0.64 ± 0.01^a^	27.6 ± 0.2^a^	57.1 ± 2.0^a^	25.3 ± 1.6^a^
Control	214 ± 2	203 ± 6^a^	3.72 ± 0.16^b^	1.06 ± 0.04^b^	44.6 ± 1.2^c^	289.3 ± 13.1^d^	149.7 ± 11.0^c^
LD	215 ± 2	228 ± 3^b^	3.86 ± 0.09^b^	1.04 ± 0.03^b^	39.3 ± 0.9^ab^	210.3 ± 2.62^b^	127.5 ± 14.2^b^
TLB	210 ± 3	234 ± 2^b^	3.33 ± 0.58^b^	1.06 ± 0.06^b^	33.2 ± 4.9^bc^	235.7 ± 1.49^c^	86.0 ± 15.6^b^

Values are means ± SE for each group of eight animals. Values with different letters are significantly different (*P* < 0.05, Duncan's multiple comparison tests). LD (*L*. *davurica*) and TLB (tolbutamide) were administered at a dose of 250 mg/kg body weight for 5 weeks.

**Table 2:** Effect of LD extract on serum TC, TG, TBARS, and liver TBARS and glycogen levels in STZ-induced diabetic rats.

Group	Serum TC	Serum TG	Serum TBARS	Liver TBARS	Liver glycogen
	(mg/dL)	(mg/dL)	(nM/mL)	(nM/mg protein)	(*μ*g/mg tissue)
Normal	73 ± 5.3^a^	80.0 ± 4.0^a^	9.01 ± 0.2^a^	1.32 ± 0.08^a^	4.46 ± 0.37^b^
Control	140 ± 10.0^c^	256.4 ± 17.5^c^	12.14 ± 0.3^b^	2.92 ± 0.06^d^	2.77 ± 0.10^a^
LD	90 ± 4.5^ab^	197.9 ± 24.4^b^	8.96 ± 0.3^a^	2.02 ± 0.07^ab^	3.92 ± 0.45^ab^
TLB	90 ± 5.3^ab^	159.9 ± 28.6^b^	11.75 ± 0.6^b^	1.90 ± 0.05^b^	3.86 ± 0.13^ab^

Values are means ± SE for each group of eight animals. Values with different letters are significantly different (*P* < 0.05, Duncan's multiple comparison tests). LD (*L*. *davurica*) and TLB (tolbutamide) were administered at a dose of 250 mg/kg body weight for 4 weeks.
